# The Role of Family Behaviors in Determining Income Distribution: The Case of South Korea

**DOI:** 10.1007/s13524-018-0670-y

**Published:** 2018-04-24

**Authors:** Inhoe Ku, Wonjin Lee, Seoyun Lee, Kyounghoon Han

**Affiliations:** Department of Social Welfare, College of Social Sciences, Seoul National University, 1 Gwanak-ro, Gwanak-gu, Seoul, 08826 Republic of Korea

**Keywords:** Income distribution, Inequality and poverty, Counterfactual decomposition, Rank-preserving exchange, Conditional reweighting

## Abstract

In this article, we examined what has contributed to the worsening income inequality and poverty between 1996 and 2011 in South Korea. We used a rank-preserving exchange method and a conditional reweighting method to assess the roles of family behaviors—including female labor force participation and family structure—characteristics of household heads, and men’s earnings. The results showed that the change in men’s earnings was a dominant factor in accounting for the increasing income inequality and poverty. The change in age and education among household heads also contributed significantly to the worsening income distribution. The change in family structure mainly affected the income disparity among lower-income families and increased poverty. The rise in women’s labor force participation improved the income distribution but not considerably. The distributional roles of family have not worked to prevent or reverse the worsening income distribution in the past few decades in South Korea.

## Introduction

Rising income inequality has become a worldwide concern and is now at the center of public discussion. The topic started to draw the attention of a few countries, such as the United States and the United Kingdom, in the 1980s and that interest later spread to countries in Europe and other regions in the 1990s (Atkinson 2015). Increasing inequality in male earnings has unequivocally been recognized as a main cause thereof, while the role of family changes has been in dispute. Because family behaviors have become an area of research interest, the effects of family structure and women’s work have been examined in many studies (for earlier studies, see Cancian et al. 1993; Karoly and Burtless 1995; Treas 1983). However, the literature faces unresolved and newly emerging issues and has yet to reach a consensus on the contributions of family changes.

Families are evolving along many dimensions. The growth of single-parent families and female work has been a focus of prior research. However, such studies have provided mixed findings on the extent and/or direction of the contributions of family changes to worsening income distribution (Cancian and Reed 1999; Martin 2006). Furthermore, changing marriage and childbearing behaviors have been generating new patterns of family formation, such as increasing numbers of single-adult families—including single-adult only families and single-parent families—and strengthened assortative mating (Breen and Salazar 2011; Schwartz 2010). Such changes impose additional complications for research on the role of family in rising inequality.

We assess the contributions of changes in family behaviors—including female labor force participation and family structure—characteristics of household heads, and men’s earnings to the deteriorating income distribution from 1996 to 2011 in South Korea (hereafter, “Korea”). We aim to contribute to the literature in three ways. First, we apply a simple version of a counterfactual decomposition method to assess contributions of family behaviors to rising income inequality. Although a counterfactual approach has been accepted in the literature, as advocated by Cancian and Reed (1999), prior research still suffers from omitted variable biases by disregarding correlations among women’s work, family structure, and other characteristics. Only a few recent studies have addressed the interdependence of women’s education, work, and marriage (Breen and Andersen 2012; Breen and Salazar 2010, 2011). We use a conditional reweighting method to remedy such biases, building on methodological innovations pioneered by DiNardo et al. (1996).

Second, we attempt to reconcile differences in findings from previous studies by providing comparable empirical results from different approaches adopted in these studies and then discussing their implications. Focusing on an evaluation of the roles of increasing women’s work, we compare findings from different methods.

Last, we extend the literature to non-Western countries because most existing studies have examined the experiences of Western countries (e.g., Chen et al. 2013; Daly and Valletta 2006; Harkness 2010). According to Jacobs (2000), family is an important contributor to improving income distribution in Asian countries. Stable and strong family ties would have helped to redistribute economic resources among family members and relatives. Yet, many aspects of family behaviors have experienced considerable changes, as exemplified in Korea. Our study of the Korean case may provide different aspects of family changes and their contributions to changing income distribution.

## Income Distribution in Korea

As in some other East Asian countries, Korea has been known for its equal income distribution as well as its fast economic growth during the industrialization period (World Bank 1993). However, during the last two decades, the landscape has changed radically. Economic growth has been faltering, and income distribution has been worsening. Available evidence suggests that in the early 1990s, Korea reached its most equal income distribution since the period of industrialization (Ku 2006). From the mid-1990s onward, however, the distribution began to worsen.

In Table 1, the first panel presents statistics on the distribution of family disposable income among households with a working-age (25–64 years) head in 1996 and 2011.^1^ The median income rose by approximately 6 % from 19.6 million Korean won (KRW) to 20.8 million KRW over the period. All the indices show a large increase in income inequality and poverty. For example, the Gini coefficient skyrocketed from 0.28 to 0.35 during such a short period. Unlike in Western countries, Korea’s income disparities widened more among lower-income families. The two percentile dispersion ratios, P90/P50 and P50/P10, started at similar levels in 1996. In 2011, P50/P10 was higher than P90/P50. Similarly, the poverty rate grew rapidly, from 8.6 % to 14.5 %.

**Table 1:** Statistics of income distribution among households with a working-aged head

Statistics	1996	2011
A. Family Income		
Median (10k KRW/year)	1,958.28	2,083.00
Coefficient of variation	0.68	0.86
P90/P10	3.42	4.96
P90/P50	1.81	2.09
P50/P10	1.89	2.37
Gini coefficient	0.28	0.35
Theil’s coefficient	0.14	0.22
Mean logarithmic deviation	0.15	0.23
Poverty rate (50 % of median) (%)	8.58	14.49
B. Family Earnings		
Median (10k KRW/year)	1,767.53	1,979.90
Coefficient of variation	0.74	0.92
P90/P10	5.02	7.88
P90/P50	1.87	2.30
P50/P10	2.68	3.43
Gini coefficient	0.33	0.40
Theil’s coefficient	0.20	0.30
Mean logarithmic deviation	0.56	0.72
Poverty rate (50 % of median) (%)	14.35	19.12
C. Men’s Earnings		
Median (10k KRW/year)	2,964.24	3,000.00
Coefficient of variation	0.78	0.99
P90/P10	6.13	9.60
P90/P50	1.84	2.40
P50/P10	3.33	4.00
Gini coefficient	0.34	0.41
Theil’s coefficient	0.23	0.32
Mean logarithmic deviation	0.84	0.91
Poverty rate (50 % of median) (%)	15.36	17.96

*Notes:* Samples are restricted to households with a working-age head (25–64 years). Data are weighted using the survey household weights. Zero and minus incomes are replaced with 0.1 in the calculation of Theil’s coefficient and mean logarithmic deviation. Family income is measured as disposable income, total income net of taxes and social insurance contributions, adjusted for differences in household size by applying an equivalence scale (square root of household size) and expressed in 2011 KRW. Family earnings are the sum of earnings of heads and spouses, adjusted for differences in household size by applying an equivalence scale (square root of household size) and expressed in 2011 KRW. Men’s earnings are earnings of male heads or spouses in households with a working-age head (25–64 years), expressed in 2011 KRW. Zero earnings are included in the analyses.

The worsening distribution of family disposable income has been largely driven by changes in earnings distribution in many industrialized countries (Organisation for Economic Co-operation and Development (OECD) 2011). This is also true of the Korean experience (Jeong 2001; Lee 2008). Inequality and poverty of equivalent family earnings, presented in the second panel of Table 1, follow a pattern similar to those of family disposable income but at a higher level.^2^ The Gini coefficient rose 21 %, from 0.33 to 0.40. The percentile dispersion ratio among the lower-income group (P50/P10) also rose, from 2.7 to 3.4. Meanwhile, the poverty rate increased from 14.4 % to 19.1 %.

The distribution of family disposable income in Korea is worse than in many developed countries in the West, whereas family earnings distribution in Korea is much better than that in most Western countries. The OECD (2011) international comparison suggested that market incomes including labor earnings are equally distributed in Korea, although disposable incomes are not due to limited welfare spending. On the other hand, the same report showed that personal earnings are distributed very unequally in Korea. For example, wage inequality was higher in Korea than in most OECD member countries in 2008 (OECD 2011).

The apparent contradiction between the equally distributed family earnings and the unequal distribution of individual labor earnings in Korea is not hard to explain. Family income distribution is determined not only by individual earnings distribution but also by grouping individuals and pooling their resources in families (Breen and Salazar 2011). The relatively equal distribution of family earnings can be explained by strong family ties in terms of family formation and work behaviors of family members in Korea, compared with those in Western countries. Family behaviors in a society can change over time. A question more relevant to our study is how family changes affect family income distribution independently from changes in personal earnings distribution. Thus, we need to look at changes in personal earnings distribution and family behaviors in Korea over the period of observation.

The bottom panel in Table 1 shows the distributions of men’s earnings in the two years, suggesting that the worsening family income distribution may be largely driven by the changes in male earnings distribution. The Gini coefficient again rose 21 %, from 0.34 to 0.41, while the poverty rate increased from 15.4 % to 18.0 %. The worsened earnings distribution is usually explained by a higher return of education due to a skill-biased technological change (Choi and Jeong 2003). Institutionally focused explanations tend to emphasize the roles of growing irregular employment and increasing wage differentials by firm size (Jung 2007; OECD 2015).

Other factors are also potential players in the changing income distribution. Above all, changes in family behavior with regard to marriage, childbearing, and women’s work may affect family income distribution by changing patterns of income pooling and income needs across families (Burtless 1999; Reed and Cancian 2012). Empirical studies have identified family structure and wives’ work as significant contributors to changing income inequality (Burtless 1999; Cancian and Reed 1998, 1999; Daly and Valletta 2006; Karoly and Burtless 1995; Larrimore 2014; Martin 2006).

Table 2 shows labor force participation rates among females living in a household headed by an adult aged 25–64; only household heads and spouses are included. The female labor force participation rate grew from 48.8 % to 53.3 %. Women heading a household had much higher rates than others, but wives also increased their market work. Increases in women’s labor force participation in our sample parallel Korean statistics on labor force participation among women aged 15–64, which suggest an increase from 49.9 % in 1990 to 55.2 % in 2012 (Yeo et al. 2013). Women’s greater labor force participation tends to be related to an improvement in their education. In fact, women’s education has rapidly improved. For example, four-year college graduation among women increased from 8.9 % in 1996 to 22.3 % in 2011. Yet, labor force participation rates do not vary much by education. The rates for college graduates increased from 50.1 % in 1996 to 56.4 % in 2011, whereas the rates for those with less than a high school education remained at 54.1 % in both years. Thus, women’s higher education may have increased the women’s labor force participation but to a much lesser degree than in most Western countries. Women’s labor force participation may have a more equalizing effect on family income distribution in Korea than in Western countries.

**Table 2:** Descriptive statistics of family characteristics

Statistics	1996	2011
Women’s Labor Force Participation		
Among female household heads or spouses	48.79	53.30
Among female household heads	82.85	86.00
Among female spouses	41.44	44.60
Family Structure	100.00	100.00
Couple with children younger than 19 years	62.34	45.95
Couple without children younger than 19 years	20.06	27.06
Headed by a single man	7.11	12.23
Headed by a single woman	10.49	14.76
(single parent)	(3.83)	(5.70)
Household Head Characteristics		
Education level	100.00	100.00
Less than high school	30.26	18.07
High school graduates	42.28	36.73
Entered university (including 1–3 years college graduates)	9.22	14.65
Four-year university graduates	18.24	30.54
Age	100.00	100.00
25–29	12.00	3.89
30–39	37.08	24.43
40–49	27.67	34.08
50–64	23.25	37.59

Changes in family structure are more conspicuous over the given period. For instance, the low fertility rate influenced family structure during recent decades: families consisting of couples and their children decreased by 16 percentage points, from 62 % to 46 %. By contrast, families of couples without children grew by 7 percentage points. Reducing marriage rates and increasing divorce rates also changed the landscape. Although the incidence of single-parent families did not significantly increase, the share of families headed by a single adult grew by 9 percentage points. Women’s rising education may have increased the proportion of single-adult families (Breen and Salazar 2010). Note that families composed of couples with or without children are much more prevalent, suggesting that families play a still stronger role in (re-)distributing resources in Korea.^3^

Characteristics of household heads show rather dramatic changes over the examined period. Education levels among heads rapidly rose. For instance, household heads who did not complete high school dropped by 12 percentage points, and household heads with a four-year college education increased as much. The improvement in education coincided with the fast aging of household heads. Rapid population aging significantly changed the age profile of household heads. The share of heads aged 50–64 increased by 14 percentage points, whereas the share of those under age 40 decreased by 21 percentage points.^4^ The concomitant rise in age and education among household heads can be explained mainly by the fact that education levels of all age groups improved during recent decades, possibly because educational improvement started early and lasted for several decades in Korea. Secondary education expanded since the 1960s. Tertiary education also started to grow in the 1970s and continued until recently.

## Data

We use data from two surveys conducted at different time points by Statistics Korea. Our data come from the 1996 National Survey of Family Income and Expenditure (NSFIE) and the 2012 Survey of Household Finances and Living Conditions (SHFLC). We use the NSFIE data because it is the most representative survey reporting detailed information on the annual income of individual members in each household during the 1990s. In addition, we use the SHFLC because it provides information on the income of families representing the whole population for the period after the 2008 financial crisis. Although the survey started recently in 2011, it is a high-quality survey and has been used for estimating statistics on income inequality, as publicly announced by Statistics Korea. The two points of time selected for our study are rather comparable in terms of business cycle. Income distribution had not started to worsen until 1996, right before the Asian economic crisis. Around 2011, Korea had already recovered from the global financial crisis, which began in 2008.

We examine the change in income distributions among households headed by adults aged 25–64. Both surveys define the head of household as a person who is the household’s main earner. Because the NSFIE does not include farmer households, we also exclude a small number of farmer households from the SHFLC for comparability. The data for analyses include 21,523 households in 1996 and 14,694 households in 2011.

Two measures of income in our analyses are family disposable income and family earnings. Because studies on inequality generally rely on family income, we analyze family income to provide comparable results. However, as we discuss later, analyzing family earnings may provide more interpretable estimates on the effects of changing family behaviors. Family disposable income is defined as total income net of taxes and social insurance contributions. Family earnings include wages, salary, and earnings from self-employment for heads and spouses. All income values are expressed in 2011 KRW, converted by the consumer price index (CPI). Family disposable income and family earnings are adjusted for family size using an equivalence scale (square root of the number of household members). Using the sample weights provided in the surveys, we adjust the data to be representative of the associated population.

## Method

### Counterfactual Decomposition Approach

This study assesses contributions of changes in men’s earnings, female labor force participation, family structure, and characteristics of household heads to the change in income distributions between 1996 and 2011. Of particular interest is whether the change in income distribution is affected by changing female labor force participation and family structure. To address this question, we use a method based on the construction of counterfactual income distributions for factors under examination. We would like to compare the hypothetical change in income distribution if, for example, female labor force participation had not changed with the actual change in income distribution if female labor force participation had changed as observed. The difference can be stated as the causal effect of the changing female labor force participation.

A traditional approach is to decompose income distribution by subgroup populations or income sources (Mookherjee and Shorrocks 1982; Shorrocks 1983; for a more recent application, see Jenkins 1995). This method provides a mechanical approach to decomposition but does not offer a natural interpretation (Biewen 2001). A basic problem is that components of the traditional decomposition have no implicit counterfactual distribution, without which decomposition results cannot be meaningfully interpreted (for a detailed discussion, see Cancian and Reed 1998). Furthermore, the traditional decomposition is confined to specific measures with particular decomposition properties. In contrast, the counterfactual distribution method adopted in this study can be used to estimate any inequality or poverty indices (Fortin et al. 2011).

Our counterfactual decomposition method is more orientated toward a nonparametrical approach. A parametric decomposition analysis of family income distribution has been conducted (Bourguignon et al. 2008). We did not opt for applying a parametric method with strong assumptions given the complexities of various factors interacting to determine family income. Following Daly and Valletta (2006), we combine two methods for obtaining a counterfactual distribution: a rank-preserving exchange of income distribution and a conditional reweighting method.

### Rank-Preserving Exchange Method

We use a rank-preserving exchange method for evaluating the contribution of changing men’s earnings, as done in previous research (Burtless 1999; Daly and Valletta 2006). This method is used to construct a counterfactual income distribution by substituting the earnings of a man of a specific rank in 2011 with the earnings of a man of a corresponding rank in 1996. In our study, we first group male earnings by 2,500 quantiles and calculate median earnings for each quantile in 1996 and 2011, respectively. Next, for men who belong to the *n*th quantile in 2011, we subtract the 2011 median earnings of the *n*th quantile from their 2011 individual earnings and add back the 1996 median earnings of the *n*th quantile.

The resulting counterfactual earnings distribution has (approximately) the same distributional characteristics as an actual 1996 earnings distribution, and the rank of each man in the earnings distribution is (approximately) preserved as observed in 2011.^5^ The difference between the actual 2011 family income distribution and the newly constructed distribution of a hypothetical family income based on the counterfactual men’s earnings shows the contribution of changing men’s earnings. This method considers a change in men’s earnings as exogenous and unconditional. This choice can be justified if men’s earnings are largely determined by labor market conditions and do not depend much on family characteristics.^6^

### Conditional Reweighting Method

We adopt a conditional reweighting method, pioneered by DiNardo et al. (1996) and applied to the decomposition of family income distribution by Daly and Valletta (2006), to assess the contributions of changes in female labor force participation, family structure, and characteristics of household heads. Our reweighting method, suggested by Biewen (2001), is simple and easy to use. Unlike DiNardo et al. (1996), who used reweighted kernel-density estimates of the income distribution before integrating them to obtain distribution indices, we directly use reweighted data to estimate such indices. This method is also more comparable with the vast literature on income distribution relying on measures calculated from unsmoothed, discrete data (Biewen 2001).

The merits of our conditional reweighting method can be easily understood in the following expository two-variable situation: female labor force participation (work or not) and family structure (single or married).^7^ Consider the case of unconditional reweighting first. To evaluate a contribution of a change in female labor force participation, we can construct a counterfactual distribution in which a share of working women is adjusted by unconditionally reweighting the data. For example, suppose that the share of working women rose from 40 % to 50 % between Times 1 and 2. We can construct a counterfactual distribution in which the share stayed at 40 % in Time 2 by reweighting working women by 40/50 and nonworking women by 60/50 in the Time 2 sample. The difference between the actual income distribution of the Time 2 sample and the counterfactual distribution can be attributable to a contribution of changing women’s labor force participation.

The unconditional reweighting method implicitly assumes that a change in women’s labor force participation is independent from a change in family structure. If female labor force participation partly depends on family structure, however, the unconditional method provides a biased estimate of a contribution made by changing women’s labor force participation by ignoring the role of changing family structure.^8^ A conditional reweighting method can avoid such an omitted variable bias (Gelbach 2016). For example, female labor force participation might have increased partially because of the increasing number of single women who were more likely to work than married women. To avoid the omitted variable bias, we need to assess the effect of changing women’s work as conditional on family structure. Suppose that the share of working individuals rose from 70 % to 75 % among single women, and the corresponding share rose from 30 % to 35 % among married women. Using the Time 2 sample, an appropriate counterfactual can be constructed by reweighting single working women by 70/75, single nonworking women by 30/25, married working women by 30/35, and married nonworking women by 70/65. The resulting counterfactual has the same conditional distribution of female work given family structure as observed in Time 1, while the marginal distribution of family structure remains unchanged as observed in Time 2. We can then evaluate the contribution of changing female work conditional on family structure by comparing income distributions before and after the reweighting.

In our study, we have three variables to be considered in the reweighting as presented in Table 2: female labor force participation (*L*); family structure (*S*); and household head characteristics (*C*), which are defined as a joint distribution of the head’s age and education.^9^ We need to specify an order of analyses for the three variables, considering relationships among the variables, in order to perform the conditional reweighting method. It seems natural to regard female work as dependent on the other two variables, to regard family structure as dependent on household head characteristics, and to regard household head characteristics as exogenously determined. Accordingly, we construct three reweighting factors to be applied to the 2011 sample: (1) a reweighting factor that adjusts a conditional distribution of female labor force participation given family structure and household head characteristics, *L*|*S*,*C*, as observed in 1996; (2) a reweighting factor that adjusts a conditional distribution of family structure given household head characteristics, *S*|*C*, as observed in 1996; and (3) a reweighting factor that adjusts an unconditional distribution of household head characteristics, *C*, as observed in 1996. Hereafter, the three reweighting factors are denoted by ψ_*L*|*S*,*C*_, ψ_*S*|*C*_, and ψ_*C*_, respectively.

It is straightforward to generate the first reweighting factor, ψ_*L*|*S*,*C*_, as explained in the preceding expository example, if we have the conditional probabilities of *L*|*S*,*C* for both years. The basic idea is to give a ratio of the 1996 conditional probability of *L* = *x* to the 2011 conditional probability of *L* = *x* as ψ_*L*|*S*,*C*_ to cases whose *L* has a value of *x* in 2011 (*x* = 0, 1). For each year, we estimate a logit model in which *L* is regressed on *S*, *C*, and their interactions, and use model estimates to get fitted values of conditional probabilities.^10^ One additional complication is that the sample includes households without female head or spouse—that is, single male households—for whom *L* is missing. We set the reweighting factor, ψ_*L*|*S*,*C*_, for single male households to 1.

Similarly, we generate the second reweighting factor, ψ_*S*|*C*_, using conditional probabilities estimated by a multinomial logit model in which *S* is regressed on *C* for each year. The third reweighting factor, ψ_*C*_, is generated in an unconditional way, using the proportions of each category of *C* for each year. Consequently, if we reweight the 2011 sample by ψ_*L*|*S*,*C*_ × ψ_*S*|*C*_ × ψ_*C*_, the joint distribution of all three variables will match that in the 1996 sample.

It is worthwhile to compare the conditional reweighting method with another counterfactual method recently proposed by Breen and Salazar (2010, 2011) and Breen and Andersen (2012). Suppose that we are interested in decomposing a change in inequality into contributions of two categorical variables, *A* and *B*. Based on the well-known Theil index decomposition by subgroup, Breen and colleagues adjusted distributions of independent variables at a point in time to match corresponding distributions at the other time point while keeping all the other components in the decomposition equation unchanged. Then, they observed the resulting change in the overall Theil index and attributed it to contributions of the independent variables.

Although this kind of counterfactual analysis using Theil index decomposition is not uncommon, Breen and colleagues contributed to the literature by devising a multivariate approach in which the effect of changing *A* can be separately accounted for. They constructed a counterfactual by adjusting the marginal distribution of *A* while keeping the marginal distribution of *B* and the association between *A* and *B* unchanged. This contrasts with our conditional reweighting method. Our method adjusts the conditional distribution of *A* given *B* while keeping the marginal distribution of *B* unchanged. Although Breen and colleagues’ method implicitly assumes that *A* and *B* do not causally affect each other, the conditional reweighting method assumes that *A* partly depends on *B* and thus that the marginal distribution of *A* will change if the marginal distribution of *B* changes. Given the possibility of a causal relationship between our independent variables, we prefer the conditional reweighting method.

### Decomposition of a Change in Family Income Distribution

By constructing counterfactual men’s earnings and obtaining the three reweights, ψ_*L*|*S*,*C*_, ψ_*S*|*C*_, and ψ_*C*_, we can now decompose the change in family income distribution. Following Daly and Valletta (2006) and Chen et al. (2013), we conduct a sequential decomposition to evaluate contributions of changes in the four factors to rising inequality and poverty. We assess the contribution of a factor by constructing a counterfactual distribution in which the factor under examination is distributed as in a hypothetical state and comparing it with a reference distribution.

We conduct a primary-order decomposition in the following sequence.

1. Calculate inequality and poverty indices using the actual 2011 family income distribution.
2. Calculate the indices using the counterfactual 2011 family income, denoted by *Y*_*C*_. The counterfactual family income equals counterfactually constructed men’s earnings plus all the income components, other than men’s earnings, of the actual 2011 family income.
3. Calculate the indices using *Y*_*C*_ reweighted by ψ_*L*|*S*,*C*_. In this counterfactual distribution, men’s earnings and female labor force participation (conditional on family structure and household head characteristics) are distributed as they were in 1996, and everything else is kept the same as observed in 2011.
4. Calculate the indices using *Y*_*C*_ reweighted by ψ_*L*|*S*,*C*_ × ψ_*S*|*C*_.
5. Calculate the indices using *Y*_*C*_ reweighted by ψ_*L*|*S*,*C*_ × ψ_*S*|*C*_ × ψ_*C*_.
6. Calculate the indices using the actual 1996 family income distribution.

Contributions of changes in men’s earnings, female work, family structure, and household head characteristics are respectively defined as the differences between 1 and 2, 2 and 3, 3 and 4, and 4 and 5. The differences between 5 and 6 are considered as a contribution of a change in the remaining unobserved factors.

Given that the factors are interrelated, the size of a contribution due to a particular factor might vary by the order of the decomposition. A sequential decomposition approach generally suffers from the same problem, and several studies have addressed this issue by conducting a decomposition in the reverse order (Chen et al. 2013; Daly and Valletta 2006; DiNardo et al. 1996). We also reverse the sequence of the decomposition to evaluate the extent to which results are sensitive to the order. Starting from the actual 2011 family income distribution, we cumulatively adjust the distribution of *C*|*S*,*L*, *S*|*L*, *L*, and men’s earnings in the listed sequence. The final counterfactual distribution in which all four factors are adjusted exactly matches the counterfactual distribution of Step 5 in the aforementioned primary-order decomposition.

## Results

### Main Results

Table 3 provides statistics on changes in family income distribution between 1996 and 2011. The first column presents statistics on total changes, and the remaining columns show sizes of each factor’s contribution and its shares in total changes (in parentheses). The contribution of unobserved factors is reported in the last column.

**Table 3:** Primary-order decomposition of changes in the distribution of family income, 1996–2011

Statistics	Total Change	Contribution of:				
		Men’s Earnings	Women’s Labor Force Participation	Family Structure	Household Head Characteristics	Residual Factor
Median	124.724	168.575	21.871	7.232	162.703	–235.656
		(1.35)	(0.18)	(0.06)	(1.30)	(–1.89)
Coefficient of Variation	0.183	0.101	–0.021	–0.007	0.100	0.011
		(0.55)	(–0.11)	(–0.04)	(0.54)	(0.06)
P90/P10	1.534	0.951	0.043	0.346	–0.006	0.201
		(0.62)	(0.03)	(0.23)	(–0.00)	(0.13)
P90/P50	0.280	0.173	0.016	0.011	0.008	0.071
		(0.62)	(0.06)	(0.04)	(0.03)	(0.25)
P50/P10	0.481	0.281	0.005	0.171	–0.012	0.035
		(0.59)	(0.01)	(0.36)	(–0.02)	(0.07)
Gini Coefficient	0.069	0.041	0.002	0.010	0.012	0.004
		(0.60)	(0.02)	(0.15)	(0.17)	(0.06)
Theil’s Coefficient	0.080	0.049	–0.001	0.008	0.020	0.004
		(0.61)	(–0.01)	(0.09)	(0.25)	(0.05)
Mean Logarithmic Deviation	0.083	0.045	–0.001	0.019	0.007	0.012
		(0.55)	(–0.01)	(0.23)	(0.08)	(0.15)
Poverty Rate (%)	5.904	3.445	0.113	2.021	–0.348	0.673
		(0.58)	(0.02)	(0.34)	(–0.06)	(0.11)

For a thorough understanding of the construction of the table, let us explain the results regarding poverty rates in detail first. As presented in the last row of Table 3, the poverty rate of family income rose by 5.9 percentage points, from 8.6 % in 1996 to 14.5 % in 2011 (for poverty rates, refer to the first panel in Table 1). When we exchange men’s earnings to make their distribution match that of 1996, the counterfactual 2011 family income exhibits the poverty rate of 11.0 %. Thus, a change in men’s earnings contributes to rising poverty by 3.4 (= 14.5 – 11.0) percentage points, which is 58 % of the total change, as presented in the second column. When we further adjust the conditional distribution of female work as observed in 1996, the poverty rate becomes 10.9 %. Accordingly, a change in female work contributes to rising poverty by 0.1 (= 11.0 – 10.9) percentage points, as shown in the third column. Contributions of the remaining factors are calculated in the same fashion and are provided in the subsequent columns.

Starting again from the first row in Table 3, we find that changes in men’s earnings and household head characteristics explain 135 % and 130 % of the total change in median income over the period, respectively. This suggests that each of the changes raised the median income by much more than the actual total change. Changes in women’s work and family structure had a relatively small influence on the rise in median. Most of the positive contributions of the four factors to rising median income, however, are cancelled out by the negative contributions of residual factors.

The other rows in Table 3 suggest that a change in men’s earnings is a primary driver of the worsening income distribution over the period, which is consistent with previous findings. It accounts for approximately 50 % to 60 % of total changes in most measures of income distribution. In contrast, the role of a change in female labor force participation is rather negligible. For most measures of inequality and poverty, it contributes to the total changes by 6 % or less, and directions of its contributions differ in sign across measures. For coefficients of variation, increasing women’s work shows slightly stronger negative association with inequality.

A change in family structure, listed in the fourth column of Table 3, has emerged as a significant contributor to the worsening distribution. For some measures that are sensitive to changes among the lower part of income distribution—such as P50/P10 ratio, P90/P10 ratio, mean logarithmic deviation, and poverty rate—sizes of its contribution range from 23 % to 36 %. This suggests that the change in family structure contributed to income dispersion among low-income families. Recall that a main feature of family structure change under examination is the growth of single-adult only families, rather than the increase in single-parent families as in Western countries.

Changes in household head characteristics substantially contributed to rising inequality for only some measures. Such changes increased the CV and Theil’s coefficient by 54 % and 25 %, respectively. Rises in age and education of household heads increased the median income, as mentioned earlier, but at the expense of higher income inequality. Note that the effects of changes in household head characteristics are the combined effects of rising age and improving education of heads. When we separately estimate the two effects, we find that the two factors exert influences in opposite directions (not shown in the table). Improved educational levels for household heads generally contributed to less inequality among the lower part of income distribution, whereas the aging of heads contributed to more inequality and poverty.

The last column of Table 3 shows that the contribution of residual factors is relatively small, compared with the net effect of our modeled factors, in accounting for the change in many measures of inequality and poverty; however, it is significant in explaining the change in the median income and, to a lesser degree, the P90/P50 ratio. The combined contribution of the four factors ranges from 75 % to 95 % of the total change in inequality and poverty, suggesting that our collection of explanatory factors include major contributors to the changes in income distribution over the study period.

Given the concern with the path-dependent nature of results from sequential analyses, Table 4 reports results from a decomposition analysis in the reverse order. These results show that contributions of the factors are qualitatively similar to the results from the analysis in the primary order. Changing men’s earnings explains approximately 50 % to 60 % of the total changes in inequality and poverty, presented in the fifth column according to sequential order, showing the largest contribution among the four explanatory factors as in Table 3. The fourth column shows that contributions of changing female work are still negligible for almost all measures. Changes in family structure again explain relatively large shares (15 % to 20 %) of increases in measures of P50/P10, mean logarithmic deviation, and poverty rate. The size of the contributions becomes smaller than in the primary-order decomposition, suggesting that part of the contribution is overlapping with a contribution related to characteristics of household heads. On the other hand, contributions of household head characteristics, considered as a first factor in the reverse order analysis, become larger for most measures.

**Table 4:** Reverse-order decomposition of changes in the distribution of family income, 1996–2011

Statistics	Total Change	Contribution of:				
		Household Head Characteristics	Family Structure	Women’s Labor Force Participation	Men’s Earnings	Residual Factor
Median	124.724	233.916	3.084	6.500	116.880	–235.656
		(1.88)	(0.02)	(0.05)	(0.94)	(–1.89)
Coefficient of Variation	0.183	0.071	–0.003	–0.009	0.114	0.011
		(0.39)	(–0.02)	(–0.05)	(0.62)	(0.06)
P90/P10	1.534	0.358	0.185	–0.017	0.807	0.201
		(0.23)	(0.12)	(–0.01)	(0.53)	(0.13)
P90/P50	0.280	0.056	–0.007	–0.001	0.160	0.071
		(0.20)	(–0.02)	(–0.00)	(0.57)	(0.25)
P50/P10	0.481	0.110	0.098	–0.007	0.244	0.035
		(0.23)	(0.20)	(–0.01)	(0.51)	(0.07)
Gini Coefficient	0.069	0.018	0.005	–0.001	0.043	0.004
		(0.27)	(0.07)	(–0.02)	(0.62)	(0.06)
Theil’s Coefficient	0.080	0.025	0.005	–0.002	0.048	0.004
		(0.32)	(0.06)	(–0.03)	(0.60)	(0.05)
Mean Logarithmic Deviation	0.083	0.018	0.013	–0.003	0.043	0.012
		(0.22)	(0.15)	(–0.03)	(0.52)	(0.15)
Poverty Rate (%)	5.904	1.122	1.207	–0.101	3.004	0.673
		(0.19)	(0.20)	(–0.02)	(0.51)	(0.11)

Overall, the results clearly indicate that the change in men’s earnings is a primary contributor to the rising inequality and poverty. Changing family structure seems to have deteriorated the position of low-income families and increased poverty. In contrast, the rise in age and education of household heads mainly contributed to inequality. Changing women’s work is not an important contributor to a change in family income distribution except for coefficients of variation.

In the decompositions of the change in family income distribution in Tables 3 and 4, interpreting contributions of the factors might be somewhat complicated because of confounding influences of income sources other than earnings of heads and spouses, such as earnings of other family members, property income, and government transfer income. One way to lessen the complication would be to focus on family earnings, defined as the sum of earnings of heads and spouses, in assessing contributions of factors under examination.

Table 5 presents results from a decomposition analysis of changes in the distributions of family earnings. In this decomposition, contributions of unobserved factors, shown in the last columns of Tables 3 and 4, are reduced for many distributional indices. This suggests that the combined explanatory power of the four factors indeed increases when we focus on family earnings. The results show some differences from those provided in Table 3. The change in men’s earnings still plays a major role in explaining the total change in family earnings distribution, although its contribution tends to be slightly smaller for many measures. On the other hand, changes in household head characteristics show larger contributions to the worsening distribution for many measures.

**Table 5:** Primary-order decomposition of changes in the distribution of family earnings, 1996–2011

Statistics	Total Change	Contribution of:				
		Men’s Earnings	Women’s Labor Force Participation	Family Structure	Household Head Characteristics	Residual Factor
Median	212.368	138.721	24.615	–40.639	129.441	–39.769
		(0.65)	(0.12)	(–0.19)	(0.61)	(–0.19)
Coefficient of Variation	0.180	0.133	–0.022	0.024	0.066	–0.020
		(0.74)	(–0.12)	(0.13)	(0.37)	(–0.11)
P90/P10	2.858	1.518	–0.322	1.425	0.388	–0.151
		(0.53)	(–0.11)	(0.50)	(0.14)	(–0.05)
P90/P50	0.426	0.224	0.034	0.058	0.028	0.082
		(0.53)	(0.08)	(0.14)	(0.06)	(0.19)
P50/P10	0.746	0.361	–0.209	0.623	0.161	–0.189
		(0.48)	(–0.28)	(0.83)	(0.22)	(–0.25)
Gini Coefficient	0.077	0.046	–0.002	0.022	0.014	–0.002
		(0.59)	(–0.03)	(0.28)	(0.18)	(–0.02)
Theil’s Coefficient	0.096	0.060	–0.007	0.027	0.020	–0.005
		(0.63)	(–0.08)	(0.29)	(0.21)	(–0.05)
Mean Logarithmic Deviation	0.161	0.051	–0.074	0.160	0.059	–0.035
		(0.32)	(–0.46)	(0.99)	(0.37)	(–0.22)
Poverty Rate (%)	4.766	2.451	–0.282	2.382	0.729	–0.515
		(0.51)	(–0.06)	(0.50)	(0.15)	(–0.11)

More noteworthy differences from the previous decomposition are detected for the role of women’s work and family structure. As such, contributions of women’s work are not trivial any more. Its negative contributions for most distributional measures indicate that the rise in women’s work improved income distribution. For P50/P10 ratio and mean logarithmic deviation—measures sensitive to changes in the lower part of income distribution—it noticeably decreased inequality. It seems that increasing women’s work mainly reduced dispersion in the lower part of the distribution. Contributions of changing family structure also show noticeable changes. Its disequalizing effect becomes much stronger, especially among lower-income families. It explains more than 80 % of changes in P50/P10 and mean logarithmic deviation and one-half of the poverty increase over the period.

However, the reverse-order decomposition shows a slightly different picture (results provided upon request). Changes in men’s earnings and household head characteristics show similar contributions. Positive contributions of changing family structure to the worsening distribution become somewhat smaller for many measures. Negative contributions of increasing women’s work become smaller in general and more notably for P50/P10 (–8 %) and mean logarithmic deviation (–16 %). Perhaps contributions of women’s work—estimated in the reverse-order analysis in which women’s work is unconditionally adjusted—incorporated disequalizing effects of household head characteristics and family structure. Overall, decompositions of a change in family earnings distribution seem to reveal contributions of women’s work and family structure, which were masked in the previous results because of influences of income sources other than earnings of heads and spouses.

### Supplementary Analysis

The decompositions of a change in family earnings distribution suggest that the rise in women’s work decreased inequality, yet the evidence is not quite strong and consistent. Previous studies did not provide consistent findings either. In the United States, some studies have found the equalizing effect of increasing women’s work (Cancian and Reed 1999; Cancian et al. 1993; Daly and Valletta 2006). Still other studies reported conflicting findings (Karoly and Burtless 1995). Some studies of income inequality in Korea have also reported the equality-enhancing effect of women’s work (Kim 2014; Lee 2008), yet other studies have not supported those findings (Chang and Lee 2013).

Varying results among many studies may be due to differences in approaches. First, a few studies (including ours) assessed contributions of increasing women’s labor force participation by a conditional reweighting method, whereas other studies used an unconditional reweighting method (e.g., Larrimore 2014). As Gelbach (2016) noted and as we mentioned earlier, unconditional methods do not control other characteristics correlated with women’s work, leading to an omitted variable bias.

Second, some studies examined contributions of increases in women’s work in terms of women’s earnings (Cancian and Reed 1998, 1999). Women’s earnings are a function of both labor supply and wage rate. If studies adjust earnings unconditionally by disregarding other characteristics related to wage rates, the contributions of women’s work may have reflected changes not only in women’s labor supply but also in their wage rates and related characteristics, even further aggravating an omitted variable bias.

Third, although some studies analyzed contributions of rising work among all women, many studies examined contributions of wives’ work among married-couple families. As Cancian and Reed (1999) noted, a focus on wives’ work may have led to a different finding.

Fourth, a few studies have examined a variety of distributional measures to assess contributions of women’s work, whereas others focused on CVs. A CV is more sensitive to changes in the upper part of income distribution than are other measures (Cowell 2011).

Given the different methodological approaches adopted across studies, we clarify their implications for the different findings by comparing results when we apply different approaches to our data. We use an unconditional reweighting for women’s labor force participation and then for wives’ labor force participation by restricting the sample to married-couple families. We also conduct another decomposition based on an unconditional adjustment for wives’ earnings.

First, we apply an unconditional reweighting method to the decomposition of changing family earnings distribution. Women’s work, family structure, and household head characteristics are adjusted, respectively, unconditionally after men’s earnings are exchanged as before. Table 6 shows that rising women’s work does not improve family earnings distribution for most measures, although it reduces mean logarithmic deviation by 20 % of the total change. Restricting the sample to couple families does not change the conclusion much either (results can be provided upon request). This finding contrasts with results from the decomposition based on a conditional reweighting method, as presented in Table 5. The unconditional method might have decreased the equalizing effects of increasing women’s work because of an omitted variable bias. We find that disequalizing effects of changes in household head characteristics decreased for many measures here when compared with the results in Table 5. Judging from this change, we suspect that contributions of changing women’s work, estimated by the unconditional method, might have incorporated part of the positive influences that changes in age and education of heads exerted on increasing inequality.

**Table 6:** Unconditional decomposition of changes in the distribution of family earnings, 1996–2011

Statistics	Total Change	Contribution of:			
		Men’s Earnings	Women’s Labor Force Participation	Family Structure	Household Head Characteristics
Median	212.368	138.721	15.680	–82.981	140.531
		(0.65)	(0.07)	(–0.39)	(0.66)
Coefficient of Variation	0.180	0.133	–0.005	0.025	0.037
		(0.74)	(–0.03)	(0.14)	(0.21)
P90/P10	2.858	1.518	0.017	1.256	0.083
		(0.53)	(0.01)	(0.44)	(0.03)
P90/P50	0.426	0.224	0.009	0.058	0.059
		(0.53)	(0.02)	(0.14)	(0.14)
P50/P10	0.746	0.361	–0.005	0.535	–0.049
		(0.48)	(–0.01)	(0.72)	(–0.07)
Gini Coefficient	0.077	0.046	–0.000	0.021	0.007
		(0.59)	(–0.00)	(0.27)	(0.08)
Theil’s Coefficient	0.096	0.060	–0.002	0.026	0.009
		(0.63)	(–0.02)	(0.27)	(0.09)
Mean Logarithmic Deviation	0.161	0.051	–0.032	0.143	–0.005
		(0.32)	(–0.20)	(0.89)	(–0.03)
Poverty Rate (%)	4.766	2.451	0.236	2.438	–0.225
		(0.51)	(0.05)	(0.51)	(–0.05)

Second, we assess the roles of increasing wives’ work by adopting a decomposition method based on another unconditional adjustment method for earnings—an approach frequently used in prior research (Cancian and Reed 1998, 1999). The widely used decomposition equation shows that rising wives’ work affects inequality by changing the share (*S*_*w*_) and inequality (${CV}_w^2$) of wives’ earnings and the correlation of spouses’ earnings (ρ_*hw*_).

$$ {CV}_f^2={S}_h^2{CV}_h^2+{S}_w^2{CV}_w^2+2{\uprho}_{hw}{S}_h{S}_w{CV}_h{CV}_w, $$

where *CV*_*k*_ is the coefficient of variation (CV) of income source *k* (*f* = family earnings, *h* = husband’s earnings, *w* = wife’s earnings), *S*_*k*_ is the share of income source *k*, and ρ_*hw*_ is the correlation coefficient between spouses’ earnings. Table 7 shows related statistics in 1996 and 2011. The CV of husbands’ earnings increased significantly as expected. The CV of wives’ earnings slightly decreased from 1.86 to 1.75 over the period, mainly because of the reduced number of wives with zero earnings, which may have an inequality-decreasing effect. Between the two years, the share of wives’ earnings increased by 3.9 percentage points. The correlation coefficient of spouses’ earnings was changed from a negative (–.08) to a positive figure (.06), suggesting potential inequality-increasing effects of changes in wives’ work.

**Table 7:** Statistics for decomposition of a change in the coefficient of variation (CV) of family earnings, 1996–2011

	1996	2011
CV		
Family earnings	0.711	0.856
Men’s earnings	0.797	0.950
Women’s earnings	1.857	1.748
Mean (10k KRW/year)		
Family earnings	2,041.227	2,675.187
Men’s earnings	1,710.302	2,136.648
Women’s earnings	330.925	538.539
Share		
Men’s earnings (%)	83.8	79.9
Women’s earnings (%)	16.2	20.1
Correlation Between Spouses’ Earnings	–0.075	0.063

Table 8 reports results from the decomposition of a change in CV between 1996 and 2011. We assess the contribution of wives’ earnings based on a CV of a counterfactual distribution. The counterfactual represents a hypothetical distribution in which the distribution of an income source (or its component such as the CV and share of wives’ earnings and their correlation with husbands’ earnings) is changed to resemble its corresponding distribution in 1996 while everything else is kept as observed in 2011. A difference from our previous analyses is that we do not actually construct the counterfactual distribution; rather, we calculate only a CV of the counterfactual distribution using related statistics in Table 7.

**Table 8:** Decomposition of a change in the coefficient of variation (CV) for family earnings, 1996–2011

	1996	2011	Men’s Earnings CV and Mean	Women’s Earnings CV	Women’s Earnings Mean	Correlation
CV	0.711	0.856	0.758	0.774	0.749	0.711
CV Change		+0.145	+0.099	–0.016	+0.025	+0.038
% of Change		100.0	+67.9	–11.0	+16.9	+26.3
	1996	2011	Women’s Earnings CV	Women’s Earnings Mean	Correlation	Men’s Earnings CV and Mean
CV	0.711	0.856	0.867	0.874	0.841	0.711
CV Change		+0.145	–0.010	–0.008	+0.033	+0.130
% of Change		100.0	–7.2	–5.2	+22.8	+89.7

The first and second columns of Table 8 show CVs of family earnings in 1996 and 2011. The change in CV is 0.145. The third column shows the CV of the counterfactual in which the CV and mean of men’s earnings are changed to resemble those in 1996 while everything else is kept as observed in 2011. The CV is 0.758—0.099 smaller than 0.856, the CV of actual family earnings in 2011. Thus, changes in the CV and mean of husbands’ earnings explain 67.9 % of the total change between 1996 and 2011. The fourth column reports that the CV is 0.774 in a new counterfactual distribution in which the CV of wives’ earnings is additionally adjusted as they were in 1996 while everything else is kept the same as observed in 2011. The change in the CV of wives’ earnings decreased the CV of family earnings by 11.0 % over the period. An additional adjustment of the mean of wives’ earnings increased the CV of family earnings by 16.9 %. Finally, the subsequent adjustment of the correlation of spouses’ earnings explains 26.3 % of the total change in CV. If the effect of increased wives’ earnings is considered as the sum of the changes in the mean and CV of wives’ earnings and their correlation with husbands’ earnings, it would increase inequality (32.1 % of total change).

When we change the order of the decomposition, the results are somewhat different. As listed in the third column in the bottom panel of Table 8, the change in the CV of wives’ earnings again decreases family earnings inequality (–7.2 % of total change). An adjustment of the mean of wives’ earnings additionally decreases inequality by 5.2 %. However, another additional adjustment of the correlation of spouses’ earnings contributes in the opposite direction by increasing inequality by 22.8 %. The net effect of increased wives’ work increases inequality by 10.3 %.

The results show that increased wives’ earnings worsen family earnings inequality over the period. If we compare these results with those for the coefficients of variation in the previous analyses, we find a pattern across findings from different approaches. Table 9 reports contributions of increasing women’s work by method of reweighting or adjustment, the definition of family income, and sample selection. We present only results generated in sequential analyses where men’s earnings are adjusted first.

**Table 9:** Contribution of increasing women’s work to a change in coefficient of variation (CV) of family income, 1996–2011

Method	Conditional Reweighting for Women’s Labor Force Participation		Unconditional Reweighting for Women’s Labor Force Participation		Unconditional Adjustment for Women’s Earnings
Definition of Family Income	Family income	Family earnings	Family earnings	Family earnings	Family earnings
Sample Selection	Women	Women	Women	Wives	Wives
% of Change in CV	–11	–12	–3	–3	+32

Contributions of increased women’s work differ across the three methods of adjustment. For instance, contributions range from –11 % (for family income) to –12 % (for family earnings) when we apply the conditional reweighting method for women’s labor force participation. When we use the unconditional reweighting method, contributions become smaller (–3 % for both the entire sample and the married-couple sample). As already shown in Table 8, the contribution is +32 % when we apply an unconditional adjustment method for wives’ earnings.

The comparison suggests that the definition of family income (i.e., family income vs. family earnings) and sample selection (i.e., women vs. wives) matter little, whereas methods of adjustment matter most. As mentioned earlier, the unconditional reweighting method may underestimate inequality-reducing contributions of increasing women’s work by wrongly incorporating inequality-increasing contributions of correlates such as changes in family structure and household head characteristics. The unconditional adjustment method for wives’ earnings even further exacerbates such an omitted variable bias by disregarding related changes in wage rate and its correlates. Arguably, our conditional reweighting method works best by reducing an omitted variable bias.

## Conclusion

After decades of industrialization, Korea reached its most equal income distribution in the early 1990s, with the income distribution continuing to worsen thereafter. In this article, we examine contributors to the worsening income inequality and poverty among working-age families since the mid-1990s in Korea. We assess the roles of family behaviors, including female labor force participation and family structure, and characteristics of household heads, along with the contribution of men’s earnings. The results confirm that the change in men’s earnings was a dominant factor in accounting for increasing income inequality and poverty between 1996 and 2011. Changes in household heads’ characteristics also contributed significantly to the worsening income distribution. Rapid population aging increased ages of household heads and contributed to more inequality and poverty. The change in family structure driven by the growth of single-adult families substantially affected income disparity among lower-income families and increased poverty. The increase in women’s labor force participation tended to equalize family income distribution, although it would be reasonable to conclude that it did not exert a large influence.

Overall, the results suggest that our counterfactual decomposition approach provides better estimates of the contributions of family behaviors than has previous research. Although the literature has used a counterfactual approach, prior research has often disregarded the interdependency between family behaviors and their correlates. We adopt a conditional reweighting method to reduce omitted variable biases pervasive in findings from previous studies. Our supplementary analyses focusing on the contributions of increasing women’s work also show that an unconditional adjustment may produce seriously biased estimates confounded with a change in correlated characteristics.

The U.S. experience since the 1950s shows that the inequality-increasing effect of changing men’s earnings was partly cancelled out by the opposing effect of increasing women’s work (Atkinson 2015; Cancian and Reed 1999; Treas 1983). Our research fails to find such a strong effect in Korea. Instead, we find that changing family structure contributed to more inequality, revealing a weakening role of family in improving income distribution. However, our study examines the contributions of changing family behaviors to a changing income distribution. An international comparison confirms that families still exert a strong equalizing effect on the distribution of market income in Korea (OECD 2011). This study shows only that changing family behaviors have not worked to prevent or reverse the worsening income distribution since the mid-1990s.

## Footnotes

1 Family income is measured as disposable income, total income net of taxes and social insurance contributions, adjusted for differences in household size by applying an equivalence scale (square root of household size) and expressed in 2011 KRW. Because different measures of inequality are sensitive to different parts of the distribution, we used several measures following some of the previous studies (e.g., Biewen 2001; Daly and Valletta 2006; DiNardo et al. 1996; Larrimore 2014).

2 In the second panel in Table 1 and throughout the article, family earnings are defined as the sum of head and spouse earnings. Earnings of other family members are excluded for an analytical purpose.

3 According to our calculation using the Luxembourg Income Study data, comparable families composed of couples (with or without children) constitute approximately 60 % in many Western countries.

4 The rapid changes in the characteristics of household heads may be overstated because of a sample selection problem in our survey data and a changing marriage pattern. First, our data exhibit a slightly faster aging process compared with the census data: the prevalence of household heads aged 25–29 decreased more rapidly, whereas that of heads aged 50–64 increased more rapidly. Education in our data also differ slightly from the census data: the proportion of those with less than high school education in 1996 is smaller, and the proportion of those with four-year college degrees in 2011 is larger in our data. Part of the reason for these differences may be that our data exclude farmer households. Farmer households, which may include older heads with low education more than nonfarmer households, constituted a larger proportion of households in the past. Second, the changes in the characteristics of household heads may be influenced by the fact that an increasing number of younger cohorts delay or forgo marriage and reside with their parents as a household member. This changing marriage pattern may have contributed to the increased proportion of older heads.

5 Some points need mentioning. First, a *median* earnings of each quantile—not an *actual* earnings of each man—in 2011 is replaced with a median earnings of a corresponding quantile in 1996. Otherwise, an earnings dispersion within each quantile will be wrongly eliminated. Second, approximately 7 % of men have zero earnings in both years. To reflect a possible change in male employment rate (Daly and Valletta 2006), we treat cases with zero earnings in the same manner as with those with positive earnings. Third, men with the same earnings (including men with zero earnings) are randomly sorted and are assigned to quantile groups according to that order. Fourth, we deliberately chose the number of quantile groups (2,500) to make the counterfactual earnings distribution resemble the actual 1996 earnings distribution as closely as possible.

6 The assumption of exogenous men’s earnings is rather strong in that male employment and wage might be somewhat correlated with female work, family structure, and household head characteristics. However, we argue that the unconditional approach is still acceptable considering that our primary focus is on contributions of female work and family structure, not on contributions of men’s earnings. After performing our main analyses, we repeated the same analysis without exchanging men’s earnings to check sensitivity of results to an adjustment of men’s earnings and found that contributions of female work, family structure, and household head characteristics do not vary much (results can be provided upon request).

7 For now, households without a female head or spouse are not considered, and family structure is dichotomized for simplicity of explanation.

8 Kollmeyer (2013) indicated that findings from some cross-national studies suffer from a bias by not controlling for family structure.

9 Initially, 16 categories were created by interacting 4 educational categories and 4 age categories. However, there were too few household heads aged 25–29 with an education level less than high school in 2011. To make conditional probability estimation feasible, this group was merged with high school graduates aged 25–29, and the remaining 15 groups were used.

10 Because we use a fully saturated specification, the procedure is equivalent to calculating conditional probabilities through descriptively tabulating *L* for every possible combination of *S* and *C* for each year.
